# Skin Microbiome Under Topical and Systemic Therapeutics in Atopic Dermatitis, a Cross‐Sectional Analysis From ProRaD

**DOI:** 10.1111/exd.70141

**Published:** 2025-08-05

**Authors:** Robin Rohayem, Matthias Reiger, Luise Rauer, Avidan Uriel Neumann, CK‐CARE‐study group, Claudia Traidl‐Hoffmann, Claudia Hülpüsch

**Affiliations:** ^1^ Institute of Environmental Medicine and Integrative Health, Faculty of Medicine University of Augsburg Germany; ^2^ Dermatology, Faculty of Medicine University of Augsburg Augsburg Germany; ^3^ Institute of Environmental Medicine Helmholtz Munich Augsburg Germany; ^4^ Christine‐Kühne Center for Allergy Research and Education (CK‐Care) Davos Switzerland; ^5^ ZIEL – Institute for Food & Health, Technical University of Munich Freising Germany

## Abstract

Atopic dermatitis is a common and chronically relapsing inflammatory skin disease. Long‐term management of the heterogeneous disease entity challenges patients and physicians globally. In our exploratory cross‐sectional study, we investigated the correlation of local and systemic therapies with skin microbial changes in patients with atopic dermatitis (AD). We cross‐sectionally evaluated the ProRaD cohort's study data between 2017 and 2019 at the Augsburg and Bonn study centres. Our analysis encompassed lesional skin microbiome swabs and medication data from 464 participants between 0 and 84 years of age. For comparative analysis, patients were grouped by disease severity. Categorisation of treatment levels was performed based on the treatment guideline for atopic dermatitis. In moderate AD, we found systemic therapy associated with a significantly lower relative abundance of *S. aureus* compared with patients receiving local treatment. However, skin microbial diversity did not significantly differ between therapeutic regimens. Furthermore, we observed a strong correlation between AD severity and relative *S. aureus* abundance in lesional skin swabs. Treatment choice, however, did not always align with disease severity, with substantial proportions of severely affected individuals receiving basic treatment only. Across all disease severities, patients receiving dupilumab tended to show a reduced *S. aureus* abundance compared to those receiving conventional immunosuppressive treatment and systemic glucocorticoids. Our findings align with recent research indicating reduced *S. aureus* abundance after systemic treatment with dupilumab, while topical anti‐inflammatory treatment alone does not seem to affect skin microbial composition. Further research is needed to elucidate the microbial–immunological interactions and their implications for AD treatment.

## Graphical abstract

Therapeutically induced skin microbiome modulation in moderate to severe atopic dermatitis. Our anaysis from the ProRaD cohort (*n* = 464) showed that patients under systemic immunomodulatory treatment and moderate atopic dermatitis (AD) posses less *Staphylococcus aureus* relative abundance on lesional skin compared to topically treated patients. We could additionally reveal a strong correlation between disease severity (SCORAD) and *S. aureus* abundance. Our findings support the microbiome modulating effect of systemic immunomodulatory treatment in moderate‐to‐severe AD.

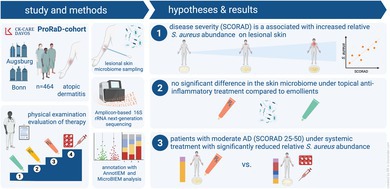

## 1 Background

Atopic dermatitis (AD, atopic eczema) is a chronic inflammatory skin disease affecting around 8% of adults and up to 20% of children and adolescents [1]. Clinical manifestations of the heterogeneous disease entity include severe itch, dry skin, erosive skin lesions, and skin lichenification. While most disease courses remain mild or moderate, severe cases can result in skin infections and sleep loss, causing heavy psychological strain on patients and their caregivers [2]. Underlying pathophysiological contributors of AD include genetic barrier dysfunction, a T_H_2‐tilted inflammatory skin phenotype, neuro‐immunological processes, and cutaneous microbial dysbiosis [3].

Microbial dysbiosis, specifically bacterial overgrowth of the skin microbiome with *S. aureus*, has been reported in the lesional skin of atopic patients for 50 years [4]. While not all patients show skin microbial dysbiosis, increased *S. aureus* abundance seems to be associated with increased disease severity [5, 6]. However, the exact contribution of the skin microbiome in developing, exacerbating, and perpetuating atopic skin disease is to be elucidated [7].

Basic treatment with emollients and proactive and reactive anti‐inflammatory topical treatment are considered standard of care for mild and moderate disease manifestations [8]. Yet, since 2017, significant advances in systemic immunomodulatory treatment have reshaped the therapeutic landscape for moderate to severe atopic dermatitis. Of note, systemic immunomodulatory agents targeting select pathophysiological pathways in AD have found their way into treatment guidelines for moderate to severe AD, demonstrating strong efficacy in reducing inflammation and disease burden [9]. However, the effect of conventional and novel treatments on the cutaneous microbiome has only been assessed through small patient cohorts and over short periods, with varying results [10, 11, 12].

The prospective cohort study ProRaD is a multi‐centre, longitudinal study investigating large patient numbers within study centres in Zurich, St. Gallen, Davos, Bonn, and Augsburg. Study visits include extended physical examination focusing on skin condition, extended anamnesis, and the retrieval of skin swabs and biomaterials [13].

## 2 Questions Addressed

This pilot study aimed to investigate the relevance of topical and systemic immunomodulatory therapies in the modulation of the skin microbiome of AD patients within the ProRaD‐cohort.

## 3 Experimental Design

### 3.1 Patient Collective

A subset of 464 participants was recruited within the prospective longitudinal cohort study ProRaD. Lesional skin microbiome swabs were obtained at a single time point during the patient's study visits between 2017 and 2019 in Bonn and Augsburg. Patients were asked not to wash, bathe, use emollients, detergents, or other substances on their skin in the 24 h preceding skin microbiome sampling. Other treatments and patient symptoms were communicated by the participants during their study visit. Treatment administered to the patient at the time of the study visit was used to classify patients into treatment levels according to the stepped‐care plan proposed through the EuroGuiDerm treatment guidelines [8, 9] (Grade 1‐Grade 4). Patients receiving basic therapy with emollients were assigned to grade 1. Patients receiving topical anti‐inflammatory treatment were assigned to grade 2 and 3, depending on the strength of the topical corticosteroid administered. Separation between grade 2 and 3 followed the grading system for topical treatment suggested by Niedner [14]. The exclusive use of topical calcineurin inhibitors was also assigned to grade 2. Patients receiving systemic treatment were assigned to grade 4. Only the treatment at the highest treatment level was considered for patient classification. Disease severity was assessed using SCORAD at the time of skin microbiome sampling. SCORAD was chosen as an established surrogate for disease severity for all analyses due to its inclusion of subjective parameters and its use in defining therapeutic boundaries throughout current guideline treatment recommendations. SCORAD values were.

Ethical clearance was approved by the ethics committee vote at the Technical University of Munich (112/16S) on 17 May 2017. Swiss‐Ethics approval was granted for Augsburg, Bonn, Davos, St. Gallen, and Zurich (EK2016‐00301).

### 3.2 Microbiome Sequencing

Microbiome analysis was conducted using amplicon‐based next‐generation 16S rRNA gene sequencing (NGS) in analogy to our previous work [15]. NGS was performed in cooperation with the Microbiome Core Facility at the Central Institute of Food and Nutrition Research (ZIEL), Technical University of Munich. Swab samples, pipeline negative controls, and water samples were sequenced using the Illumina MiSeq platform, utilising 2 × 300 bp paired‐end reads. The MiSeq Reagent Kit v3 600 cycles was applied following the manufacturer's guidelines. Data processing, visualisation, and exportation were executed using the instrument's proprietary software, Illumina Software MiSeq Reporter and Illumina Sequence Analysis Viewer. Denoising of sequences was conducted through the implementation of DADA2 [16], and the resulting amplicon sequence variants (ASVs) were annotated using our in‐house‐developed AnnotIEM [17] algorithm. An assessment of sequencing depth sufficiency was performed by correlating the read number and the number of distinct ASVs, aiming to ascertain adequate sequencing depth.

### 3.3 Taxonomic Analysis

NGS microbiome taxonomic analysis was performed using MicrobIEM. MicrobIEM is a toolset developed by our group that facilitates decontamination, quality control, and fundamental analysis of the microbiome using 16S rRNA amplicon sequencing data. It is embedded within the R Studio statistical software package [18]. Graphical visualisation and statistical analysis were performed using GraphPad Prism v.10.

A total of 1077 samples from 582 study subjects were analysed. After methodical quality control, only 974 samples remained. Of these, 889 samples came from participants diagnosed with AD. Of these, 721 swabs were taken from lesional locations. After excluding samples with unclear metadata and excluding duplicate swabs from the same subject, 464 microbiome samples remained for further analysis. Statistical analysis and visualisation were performed using GraphPad Prism 10.

## 4 Results

### 4.1 Patient Characteristics

The analysed cohort consisted of participants ranging from 0 to 84 years, with a median age of 31 years. While the age of the participants approximated Gaussian distribution, it showed significant peaks for children and adolescents. The distribution of biological sex varied considerably among treatment groups, with more female than male participants enrolled (Table 1).

**TABLE 1:** Study population characteristics and systemic treatment agents.

	Treatment level			
	I	II	III	IV
Characteristics *n* = 464^a^	Basic treatment (*n* = 193)	Topical anti‐inflammatory Grade 2 (*n* = 48)	Topical anti‐inflammatory Grade 3 (*n* = 187)	Systemic immuno‐modulatory (*n* = 36)
Age, median (min; max)	31 (0; 80)	35 (1;76)	33 (0;84)	51 (5;84)
Biological sex				
Male (in %)	73 (38%)	17 (35%)	84 (45%)	22 (61%)
Female (in %)	120 (62%)	31 (65%)	103 (55%)	14 (39%)
SCORAD – mean (SD)	33.5 (20.1)	36.2 (13.4)	41.1 (18.6)	42.3 (21.2)
EASI – mean (SD)	7.5 (9.6)	6.0 (5.1)	12.3 (12.6)	13.3 (13.3)
**Systemic treatment**				Patients (swabs)
Prednisolone (p.o.)				20 (32)
Cyclosporine A (p.o.)				4 (8)
Dupilumab (s.c.)				5 (8)
Azathioprine (p.o.)				2 (3)
Methotrexate (s.c./p.o.)				3 (4)
excluded^a^				2

^a^11 patients were later excluded from analysis due to missing or unclear data. Treatment levels were defined according to the guideline for treating atopic dermatitis [8].

Participants reported both chronic‐constant and recurrent AD disease courses in equal proportions. Most participants were moderately affected (~43.9%), with SCORAD values ranging between 25 and 50. Based on current medication use, most participants were classified as receiving basic or topical anti‐inflammatory treatment (treatment levels 1–3). Eight percent of the patients reported ongoing systemic immunomodulatory treatment (treatment level 4). Despite the general trend of higher therapeutic regimens with increasing disease severity, many patients with severe AD reported solely relying on basic therapy (Table 1).

### 4.2 Increase of *S. aureus* Correlates With Disease Severity

Before assessing therapeutic interventions' influence on the microbiome, an examination of potential confounders was carried out. No correlation was found between the relative abundance of *S. aureus* in lesional samples and age or gender (data not shown). However, a strong correlation was identified between more severe skin disease and higher *S. aureus* relative abundance (Appendix S1).

**FIGURE 1:**
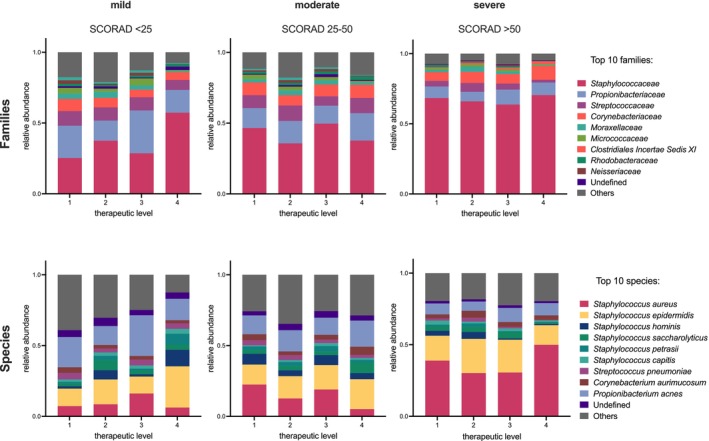
Taxonomic distribution of the ten most abundant prokaryotic families and species in lesional cutaneous microbiome swabs from mildly (SCORAD < 25; *n* = 129), moderately (SCORAD 25–50; *n* = 204), and severely (SCORAD > 50; *n* = 126) affected patients. Therapeutic regimens were assessed individually based on atopic dermatitis severity to avoid confounding. A separate analysis of the correlation between disease severity and *S. aureus* abundance is provided in Appendix S1.

Distinct differences in the skin microbiome were evident across AD severity levels (Figure 1). To minimise bias, patients were grouped by mild (SCORAD < 25), moderate (SCORAD 25–50), and severe (SCORAD > 50) disease. An increasing dominance of *Staphylococcus* species, particularly *S. aureus* , was observed in taxonomic analysis with worsening skin conditions (Figure 1). As *S. aureus* abundance increased, we found that species, such as *Cutibacterium acnes* and coagulase‐negative staphylococci, such as *S. epidermidis* and *S. hominis* , decreased in relative abundance (Figure 1).

**FIGURE 2:**
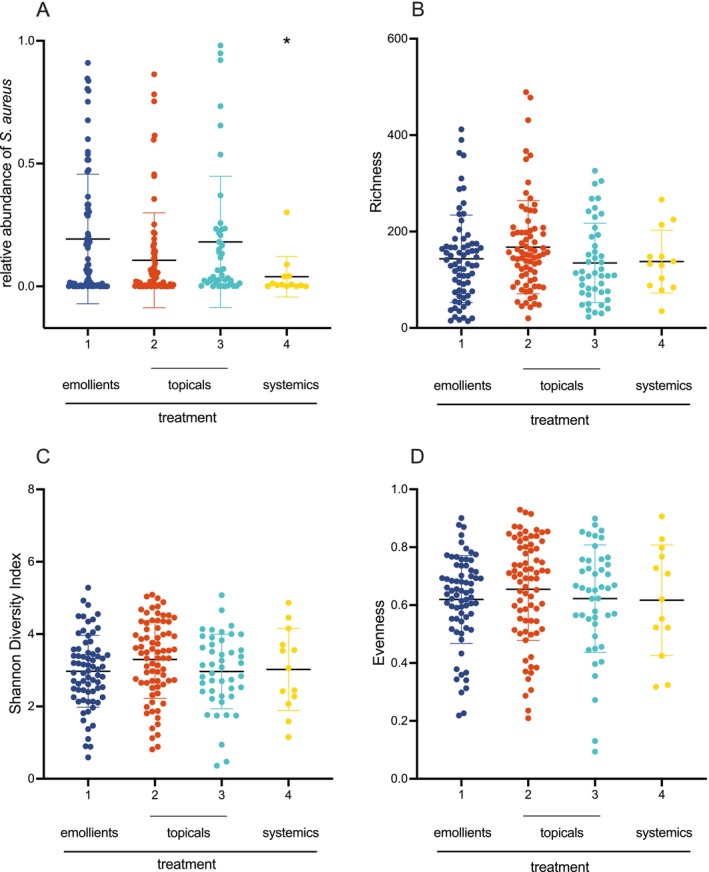
Relative abundance of *S. aureus* (A) and α‐diversity (B–D) of lesional swabs of the skin microbiome across different therapeutic regimens in patients with moderate atopic dermatitis (SCORAD 25–50; *n* = 204). Diversity measures include Richness (B), Evenness (D), and Shannon Diversity Index (C), with mean values and standard deviations shown. Each point represents a lesional skin swab from a participant, classified by therapeutic regimen. Statistical analysis of group differences was performed using the Kruskal‐Wallis test, with significance accepted at *p* < 0.05 (pA = 0.02 (*); pB = 0.17 (ns); pC = 0.15 (ns); pD = 0.46 (ns)). For confirmation of differences in relative abundance between topical and systemically treated patients, additional statistical testing was performed using one‐tailed Man–Whitney *U* testing between group 1–3 and group 4 (*p* = 0.027).

Among patients with moderate AD receiving systemic immunosuppressive therapy (therapeutic regimen 4), a significant reduction in *S. aureus* was observed compared to those on topical therapies (therapeutic regimens 2 & 3) or basic treatment (therapeutic regimen 1) (Figure 2). However, measures of microbial diversity (Richness, Evenness, Shannon Diversity Index) showed no significant differences between treatment groups. Likewise, bacterial β‐diversity showed no significant differences across therapy groups (Appendix S3).

Patients receiving systemic treatment comprised a heterogeneous group with treatments of differential mechanisms of action and effectiveness, forming the highest escalation step in the current stepped‐care treatment approach. A more detailed comparison of systemic therapies was made despite the small patient numbers (*n* = 36) receiving these treatments. The skin microbiome of participants receiving biologics exhibited overall low relative *S. aureus* abundance compared to participants receiving systemic corticosteroids, which displayed more heterogeneous results and higher *S. aureus* levels (Appendix S2).

## 5 Conclusion and Perspectives

Our study underscores the pivotal role of *S. aureus* in disrupting the skin microbial equilibrium in individuals with atopic dermatitis (AD). By examining participants within the ProRaD cohort, we demonstrated a robust positive correlation between AD severity, as quantified by SCORAD scores, and the relative abundance of *S. aureus* in lesional skin. Our findings substantiate those of prior studies, which describe bacterial overgrowth of *S. aureus* as a pivotal factor in reducing microbial diversity in AD [6, 19]. Furthermore, our results lend further support to the hypothesis that *S. aureus* colonisation may exacerbate skin barrier dysfunction, thereby acting as a crucial pathophysiological co‐factor in the pathogenesis of barrier impairment, inflammatory immune dysregulation, and imbalance of the skin microbiome [20, 21].

The comparative analysis of different therapeutic interventions revealed no statistically significant alteration in skin microbial diversity or relative abundance of *S. aureus* in patients treated topically with anti‐inflammatory agents. This finding is consistent with the results of other studies [11, 22, 23] that call into question topical corticosteroids' ability to reinstate skin microbial balance. However, these findings remain controversial as opposing observations were made in studies evaluating short‐term topical anti‐inflammatory treatment effects on the skin microbiome [24, 25]. This discrepancy may in part be explained through short‐term modulation of skin barrier properties during topical anti‐inflammatory treatment. Additionally, skin microbial modulation through topicals heavily depends on the composition and potential antimicrobial activity of the administered substance [26]. To create solid evidence for skin microbial modulation through topicals, further high‐quality trials and grouped comparisons by antimicrobial mode of action will be needed [23, 27]. To date, the exact role of skin microbiota in the pathogenesis of atopic dermatitis remains controversial [28]. In our study, we observed a significant reduction in *S. aureus* relative abundance in moderately affected participants who received systemic treatment compared to those receiving topical treatment. An in‐depth analysis of systemic treatment by mode of action demonstrated a trend towards a decrease in the abundance of *S. aureus* in patients undergoing treatment with dupilumab compared to other systemics (App. S1). Patients receiving systemic corticosteroids exhibited a more heterogeneous response and varied in skin microbial dysbiosis. Our cross‐sectional analysis hints that with more data on patients undergoing systemic treatment becoming available, statistical power could be increased in future analyses of the collective. As earlier studies have shown, demographics can act as significant confounding factors; therefore, in future studies, more demographic data would prove useful in the interpretation of the results [29].

Our findings indicate the efficacy of T_H_2‐directed systemic interventions in reducing *S. aureus* overgrowth in lesional skin. This effect can be explained through the reinstating effect of T_H_2‐blocking on anti‐microbial peptide (AMP) production, as observed in other studies [10, 30, 31, 32]. Therefore, we believe that targeted systemic T_H_2‐directed substances should be considered to directly contribute to the cutaneous microbial equilibrium. In clinical practice, effectively reducing *S. aureus* in AD patients' lesional skin areas through targeted treatments with biologicals like dupilumab has been shown to reduce incidences of secondary infections and help improve clinical outcomes [33]. These effects may be critical for the affected individuals, given persistent challenges in AD management through conventional topical treatments that are insufficiently preventing recurring skin infections [10].

In summary, our results encourage more integrated therapeutic approaches incorporating early disease modification through systemic interventions for moderate to severe AD cases.

However, the interpretation of our study underlies several limitations. One of the most notable limitations stems from the cross‐sectional pilot study design. While correlations between disease severity, microbial imbalance, and therapeutic effects were identified, the results remain descriptive as the cross‐sectional analysis does not allow the determination of causal relationships. Consequently, longitudinal evaluations of the ProRaD cohort will be needed to sufficiently illustrate the long‐term effects of local and systemic therapies on the cutaneous microbiome. While the investigated cohort reflects a heterogeneous patient collective, the generalisability of the findings remains limited, mainly due to the potential selection bias of a voluntary central European atopic dermatitis study population [6]. Treatment data were assessed shortly after the introduction of dupilumab as a systemic treatment. Repeating the analysis with more recent data would show an increased proportion of moderately and severely affected patients receiving systemic treatment, lending more statistical power to the claim of a microbiome modulating effect from systemic treatment [34]. Further limitations include methodological aspects. While 16S‐rRNA amplicon‐based next‐generation sequencing delivers a detailed image of the prokaryotic kingdom, other commensals of the skin microbiome remain undetected. These include fungi, viruses, and phages, which interact with the host and skin bacteria and thus may play an important role in AD pathogenesis [20, 35]. For a more holistic approach, future studies could benefit from incorporating whole metagenome shotgun sequencing (WMS) to capture the entire skin microbial ecosystem and allow for microbial differentiation down to strain level. A longitudinal design, an increase in systemically treated patients within the cohort, and strain level differentiation in microbiome evaluations could be meaningful in future evaluations of the cohort.

In conclusion, our pilot study underscores the complex relationship between the skin microbiome, immune dysregulation, and barrier dysfunction in AD. Our early findings hint that T_H_2‐targeted therapies may offer the dual benefit of reducing inflammation and restoring microbial homeostasis. Thereby, they could potentially prevent AD exacerbation and reduce the risk of secondary infections. However, whether skin microbial imbalance is merely a symptom or causative of worsened skin conditions in AD remains to be elucidated [7]. Future longitudinal observations are needed to better understand the immuno‐microbial relationship in AD and other inflammatory skin diseases.

## Author Contributions

Robin Rohayem performed conceptualisation, formal data analysis, investigation, writing the original draft, and writing – review and editing. Claudia Hülpüsch contributed to conceptualisation, methodology, project management, writing – review and editing, investigation, and formal analysis. Luise Rauer, Avidan Neumann, and Matthias Reiger were involved in methodology, writing – review, and editing. Claudia Traidl‐Hoffmann contributed to project management, writing – review and editing, and funding acquisition.

## Conflicts of Interest

Prof. Dr. med. Traidl‐Hoffmann reports institutes grants from Sebapharma, Germany, Beiersdorf, Germany, L'Oreal, Germany; personal fees from Novartis, Germany, Sanofi, Germany, Lilly pharma, Germany, grants and Töpfer GmbH, Bencard, Germany, Danone nutricia, Lancome, Germany, Loreal, Germany, outside the submitted work—Dr. med. Matthias Reiger reports personal fees from Novartis, Germany, Reviderm Germany, Bencard Allergy and La Roche Posay outside the submitted work. Dr. rer nat. Claudia Hülpüsch reports personal fees from Reviderm, Germany, and Sebapharma, Germany, outside the submitted work. Dr. med. Robin Rohayem reports personal fees from Novartis outside the submitted work.

## Supporting information


**Appendix S1:** exd70141‐sup‐0001‐AppendicesS1‐S3.docx.

## Data Availability

The authors confirm that the data supporting the findings of this study are available from the corresponding author upon reasonable request.
