# Supplementary material for: Skin Microbiome Under Topical and Systemic Therapeutics in Atopic Dermatitis, a Cross‐Sectional Analysis From ProRaD
Source: Exp Dermatol. 2025 Aug 5;34(8):e70141. doi: 10.1111/exd.70141 (PMC12323296; doi:10.1111/exd.70141)
Supplement: Supplementary file 1 — Appendix S1: exd70141‐sup‐0001‐AppendicesS1‐S3.docx. [file EXD-34-e70141-s001.docx]

## Appendix


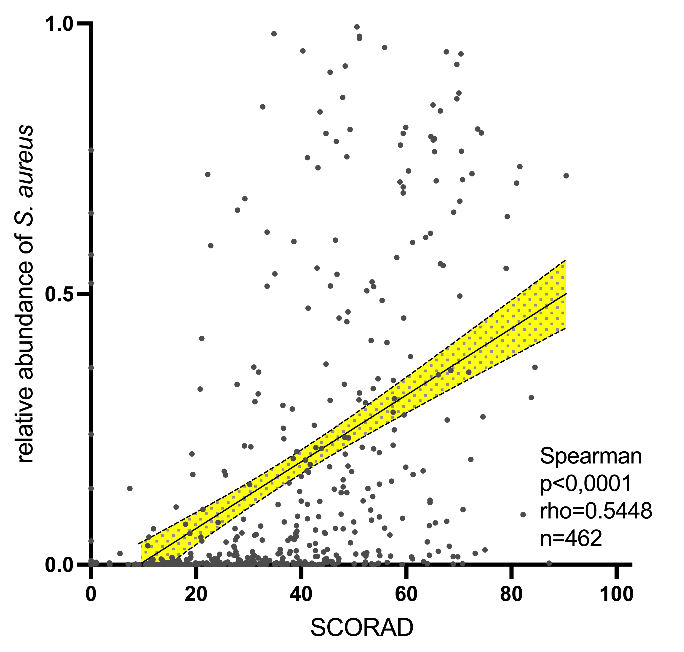


Appendix 1: Correlation of SCORAD and relative *S. aureus* abundance. SCORAD values of lesional samples (n=462) at study visit are depicted. Each point represents the score and relative *S. aureus* abundance from lesional skin swabs of a participant. The shaded area indicates the 95% confidence interval around the regression line. Statistical analysis was performed using Spearman’s rank correlation for non-normally distributed data, with a solid positive correlation defined as rho >0.5.


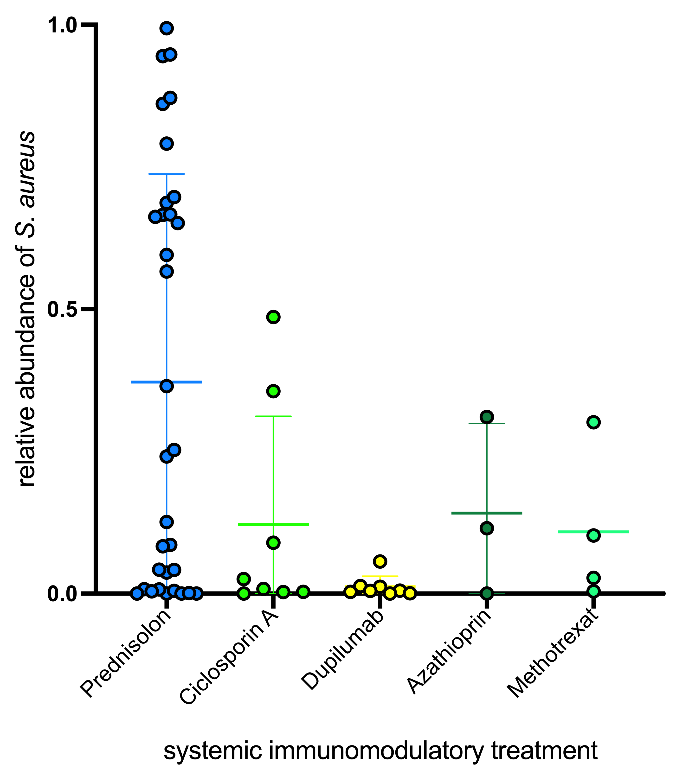


Appendix 2: Relative *S. aureus* abundance in lesional swabs (n=55) from patients on systemic immune-modulatory therapy (n=36). The relative abundance of S. aureus for each individual and the mean and standard deviation are shown. Both on- and off-label therapies for atopic dermatitis were included at the time of study enrollment.


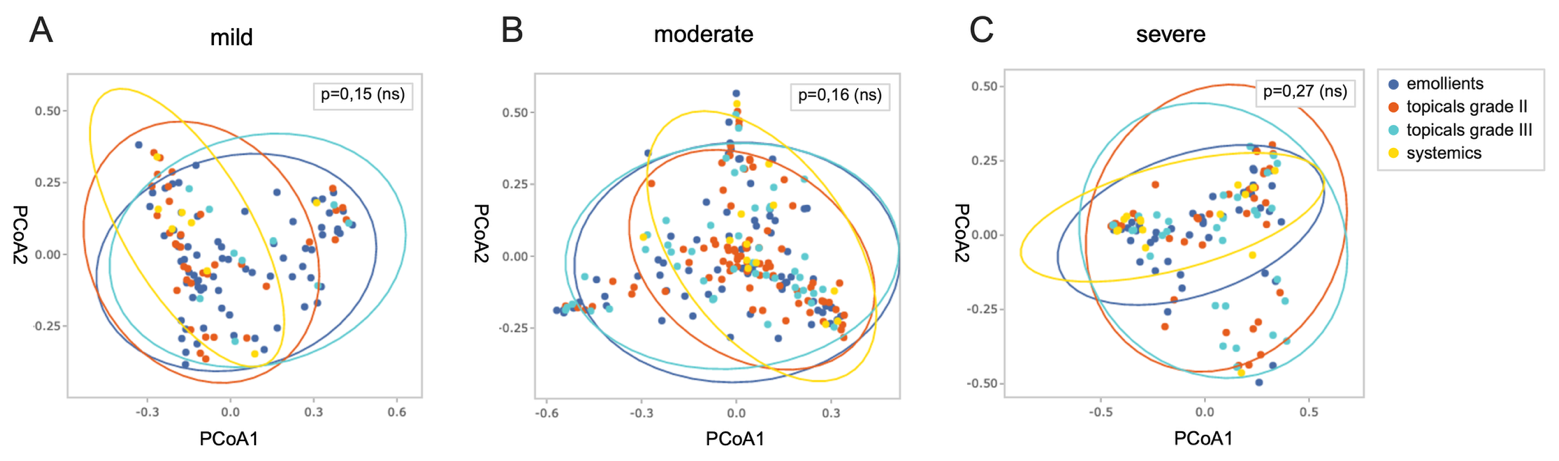


Appendix 3: Beta diversity analysis of lesional cutaneous microbiome swabs sampled from mildly (SCORAD<25; n=129), moderately (SCORAD 25-50; n=204), and severely (SCORAD>50; n=126) affected patients. Similarities between treatment regimens were analysed separately for each severity index. PCoA performed visualisations on Bray-Curtis dissimilarities. P-values are derived from PERMANOVA tests with 500 permutations. Statistical significance of differences was assumed for (p<0.05).
